# Characterization of the c.793-1G > A splicing variant in *CHEK2* gene as pathogenic: a case report

**DOI:** 10.1186/s12881-019-0862-3

**Published:** 2019-07-26

**Authors:** Konstantinos Agiannitopoulos, Eirini Papadopoulou, Georgios N. Tsaousis, Georgia Pepe, Stavroula Kampouri, Mehmet Ali Kocdor, George Nasioulas

**Affiliations:** 1 Genekor Medical S.A, Athens, Greece; 2 Eylul Universitesi Hastanesi, Izmir, Turkey

**Keywords:** *CHEK2*, Splicing variant, Next generation sequencing, RNA analysis

## Abstract

**Background:**

*CHEK2* is involved in the DNA damage repair response Fanconi anemia (FA)-BRCA pathway. An increased risk for breast and other cancers has been documented in individuals who carry a single pathogenic *CHEK2* variant. As for other genes involved in cancer predisposition, different types of pathogenic variants have been observed, including single nucleotide variations, short insertions/deletions, large genomic rearrangements and splicing variants. Splicing variants occurring in the splicing acceptor or donor site result in alternative mature mRNA produced and can cause intron retention, exon skipping, or creation of alternative 3′ and 5′ splice site. Thus, the pathogenicity of this type of alterations should always be explored experimentally and their effect in the mRNA and consequently the protein produced, should be defined. The aim of this study was the delineation of the effect of a splicing variant in the *CHEK2* gene.

**Case presentation:**

A healthy 28-year-old woman with a family history of breast and ovarian cancer was referred for genetic testing. The variant c.793-1G > A (rs730881687) was identified by Next Generation Sequencing (NGS) using a solution-based capture method, targeting 33 cancer predisposition genes (SeqCap EZ Probe library, Roche NimbleGen). Experimental analysis in patient-derived leukocytes using RT-PCR of mRNA followed by cDNA sequencing revealed the deletion of one base from the alternative transcript created (r.793del). This resulted in a frameshift leading to premature termination codon within exon 7 (p.(Asp265Thrfs*10)).

**Conclusions:**

This finding suggests that the *CHEK2* splicing variant c.793-1G > A is a deleterious variant. Our case shows that RNA analysis is a valuable tool for uncharacterized splice site variants in individuals referred for testing and facilitates their personalized management.

## Background

Germline *CHEK2* pathogenic variants have been associated with breast, prostate, colorectal, gastric, thyroid, bladder and kidney cancer [1]. *CHEK2* pathogenic variants such as the c.1100delC variant [2] are associated with breast cancer and have been characterized. In addition, other *CHEK2* variants, such as c.470 T > C, p.(Ile157Thr) and c.1283C > T, p.(Ser428Phe), are characterized as of low penetrance for breast cancer [3].

Nowadays, Next Generation Sequencing (NGS) technology has allowed multi-gene panel analysis which is used in clinical practice for the identification of individuals with an inherited predisposition to cancer. Usually, the majority of genes analyzed in such panels is included in guidelines and have clinical management [4].

The type of pathogenic variants identified using NGS methodology include single nucleotide variations (SNV), short insertions/deletions (Ins/Del), large genomic rearrangements (LGR) and splicing variants. All these alterations usually occur in tumor suppressor genes and are related to increased risk of cancer development. The classification of the variants identified in a five-tier classification system is done using the American College of Medical Genetics and Genomics (ACMG) Laboratory Quality Assurance Committee guidelines [5]. According to this system a variant can be classified as pathogenic, likely pathogenic, variant of uncertain significance, likely benign and benign. The assignment of a variant in the pathogenic class requires the presence of both experimental and in silico analysis that provide multi-level evidence for a major impact of the alteration in the protein’s function.

Splicing variants occurring in the splicing acceptor or donor site result in alternative mature mRNA production [6] and can cause intron retention, exon skipping, or creation of alternative 3′ and 5′ splice site, resulting in the production of a disrupted or non-functional protein. The detection of such variants provides strong evidence of pathogenicity based on the ACMG guidelines, but their effect can be also investigated by in silico analysis that should be confirmed either at the mRNA or the protein level.

In our case, we report the functional characterization of a *CHEK2* variant located in intron 6. The variant c.793-1G > A alters the wild type acceptor site and activates a cryptic acceptor site one nucleotide base downstream, creating a new transcript with a premature translation stop codon 10 amino acid residues later. This is expected to lead in an absent or disrupted protein product.

## Case presentation

### Patient

A 28-year-old healthy woman, with a family history of breast and ovarian cancer, was referred to our laboratory for multigene testing. Our proband was informed about the significance of molecular testing, provided information about her personal and family history and signed an informed consent form prior to molecular genetic testing and permission for the anonymous use of her data for research purposes and/or scientific publications.

### Gene testing

Genomic DNA was extracted from peripheral blood leukocytes using MagCore® Genomic DNA Whole Blood Kit (RBC Bioscience) according to the manufacturer’s instructions.

The analysis of genes involved in hereditary cancer predisposition was performed using a solution-based capture approach. Targeted NGS was performed with a panel of 33 genes (Roche NimbleGen SeqCap EZ Choice) [*APC* (NM_000038.5)*, ATM* (NM_000051.3), *BARD1* (NM_000465.2), *BMPR1A* (NM_004329.2), *BRCA1* (NM_007294.2)*, BRCA2* (NM_000059.3)*, BRIP1* (NM_032043.2), *CDH1* (NM_004360.4)*, CDK4* (NM_000075.3)*, CDKN2A* (NM_000077.4)*, CHEK1 (*NM_001114121.2), *CHEK2* (NM_007194.3)*, EPCAM* (NM_002354.2)*, MEN1* (NM_000244.3)*, MLH1* (NM_000249.3)*, MRE11* (NM_005591.3)*, MSH2* (NM_000251.2)*, MSH6* (NM_000179.2)*, MUTYH (*NM_001128425.1)*, NBN* (NM_002485.4)*, NF1* (NM_000267.3)*, PALB2* (NM_024675.3)*, PMS2* (NM_000535.5)*, PTEN* (NM_000314.4)*, RAD50* (NM_005732.3)*, RAD51B* (NM_133509.3), *RAD51C* (NM_058216.2), *RAD51D* (NM_002878.3), *RET* (NM_020975.4)*, SMAD4* (NM_005359.5)*, STK11 (*NM_000455.4)*, TP53* (NM_000546.5)*, VHL* (NM_000551.3)]. The sample preparation was performed according to the manufacturer’s instructions in the SeqCap EZ Choice Library User’s Guide (Roche NimbleGen). Sequencing was carried out using the Miseq Illumina NGS technology and sequence changes were identified and interpreted in the context of a single clinically relevant transcript using the commercially available software suite SeqNext (JSI medical systems GmbH, Germany).

### Variant classification and bioinformatics analysis

The clinical significance of variants was further examined using standards and guidelines for the interpretation of sequence variants recommended by the ACMG Laboratory Quality Assurance Committee and the Association for Molecular Pathology (AMP) [5]. The impact of missense substitutions on protein function or structure was analyzed using computational (in silico) predictive algorithms combined with the ensemble mutational impact score of MetaSVM [7]. The effect on splicing was computationally examined using Human Splicing Finder [8].

### RNA analysis

Total RNA was extracted from peripheral blood lymphocytes using Trizol (Invitrogen, Paisley, UK) following standard protocol. cDNA was synthesized using SuperScript™ VILO™ cDNA Synthesis Kit (Thermo Fisher Scientific) as described by the supplier. The resulting cDNA was amplified with the *CHEK2* specific primers CHEK2X5F_RNA:5′-ACATCATGTCAAAAACTCTTGGAA-3′ and CHEK2X8-9R_RNA: 5′-CCCCTTCCATCAATTCCAAAACAA-3′ and the PCR-products were purified using NucleoFast® 96 PCR Clean-up kit (Macherey-Nagel GmbH and Co., Düren, Germany). The purified PCR product were used for each sequencing reaction, which was performed using the BigDye® Terminator v1.1 Cycle Sequencing kit (Applied Biosystems, Foster City, CA, USA) and Sequencing reaction products were purified prior to electrophoresis using the Montage™ SEQ96 Sequencing Reaction kit (EMD Millipore Corp., Billerica, MA, USA) and sequenced using an Applied Biosystems 3130 Genetic Analyzer (Applied Biosystems).

## Results

**Fig. 1:**
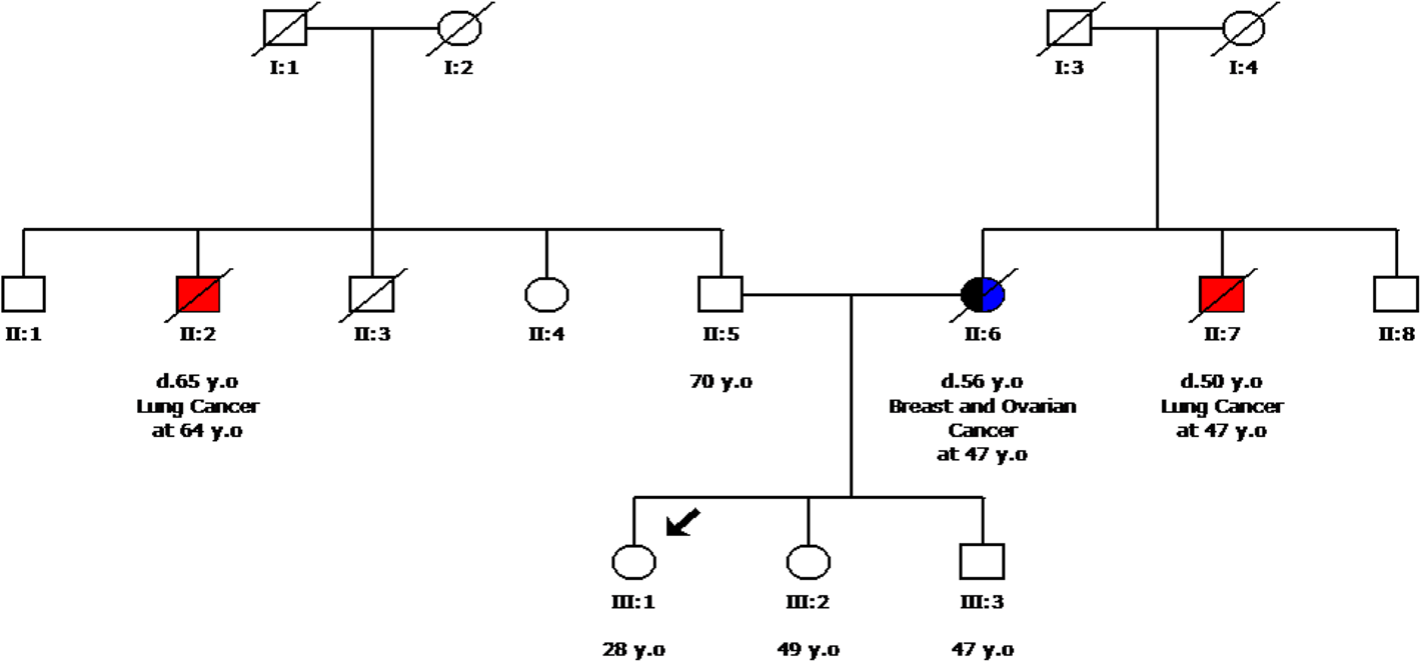
Pedigree of the proband’s family. y.o, years old; d, died. Black, blue and red colors represented breast, ovarian and lung cancer, respectively

**Fig. 2:**
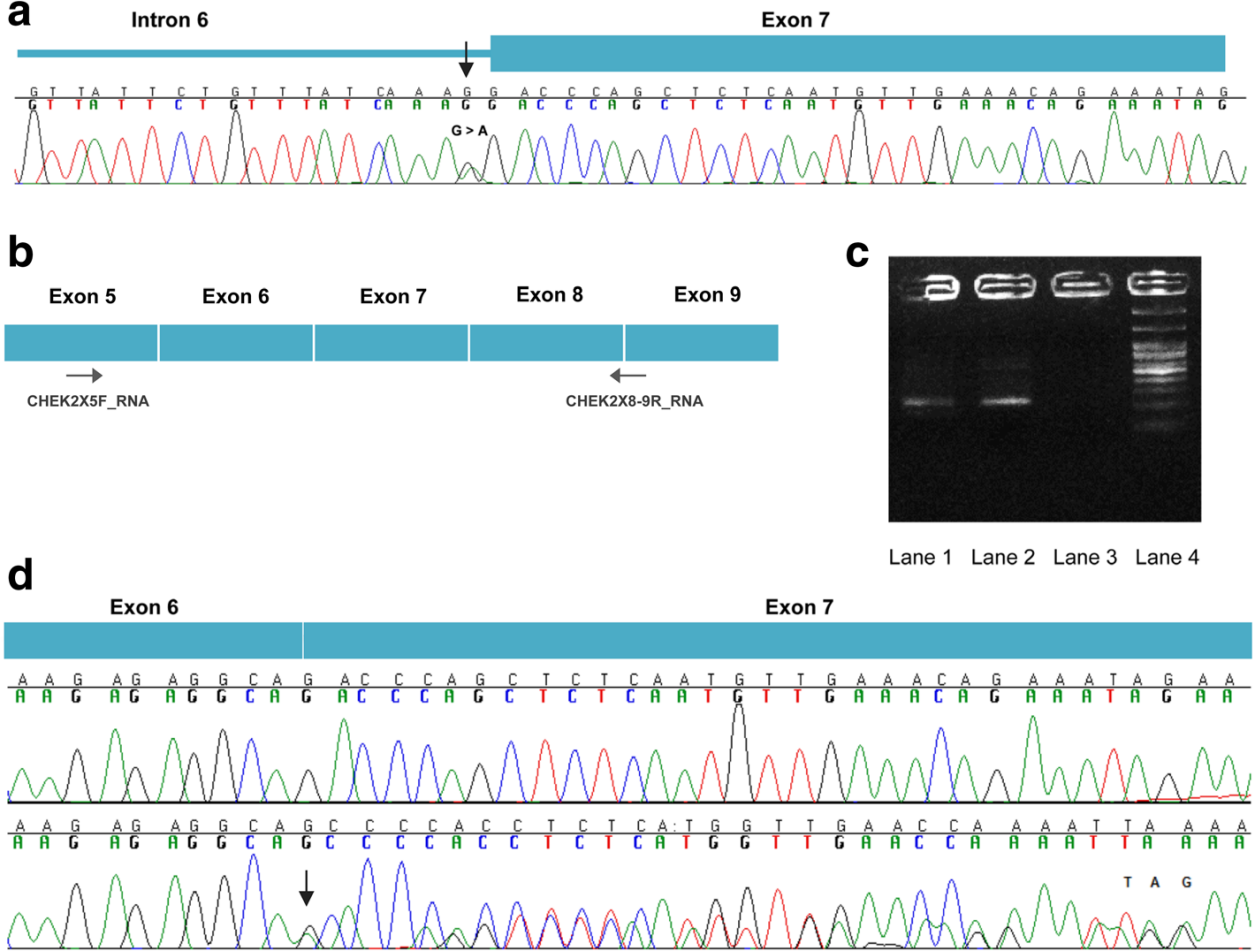
**a** Sequencing analysis of genomic DNA from the proband carrying the c.793-1G > A variant in the *CHEK2* gene. **b** Binding sites of the primers on the cDNA of *CHEK2* **c** RT-PCR electrophoresis products on 3% agarose gel. Lane 1: proband’s sample with the splicing variant, Lane 2: normal sample, Lane 3: negative control, Lane 4: 1000 bp DNA ladder (New England Biolabs). **d** Sequencing analysis of the proband’s cDNA showing the frameshift of the variant and the generation of a premature translation stop signal (TAG) 10 amino acid residues later (bottom panel) compared to the sequencing analysis of a wild type sample (top panel)

The proband was referred to genetic counseling, since her mother was diagnosed with breast and ovarian cancer at a young age (Fig. 1). Our analysis using NGS technology identified the variant c.793-1G > A (rs730881687) in the *CHEK2* gene, in heterozygosity. This alteration was a replacement of the last nucleotide base in intron 6 of the *CHEK2* gene. This finding was confirmed by Sanger Sequencing using the following forward and reverse primers (5′-TCAGGCAGCCTTGAGTCAAC-3′ and 5′- CAGCTAAATGACAGCTAGGC-3′ respectively) as described previously [9] (Fig. 2a). This particular location is strictly conserved in human and splice donor and acceptor site variants typically lead to loss of protein function [10]. Furthermore, loss-of-function variants in *CHEK2* are known to be pathogenic [11]. No other pathogenic/likely pathogenic variants were identified in the remaining 32 genes that were analyzed.

In silico analysis predicted that this change is disrupting the wild type acceptor site and is activating an intronic cryptic splice acceptor 1 bp downstream, creating a frameshift. The prediction was confirmed by RNA analysis, (Fig. 2b, c, d) showing that the c.793-1G > A variant affects splicing by creating an alternative splice site 1 bp downstream (r.793del) which results in a frameshift effect and the generation of a premature translation stop signal 10 amino acid residues later and is predicted to result in a truncated protein (p.(Asp265Thrfs*10)).

## Discussion and conclusions

The variant c.793-1G > A identified in the proband tested has been previously reported in an individual affected with breast cancer [4] and in individuals who underwent genetic tests for hereditary cancer risk [12]. The variant database ClinVar contains entries for this variant (rs730881687) where it is listed as likely pathogenic and pathogenic (https://www.ncbi.nlm.nih.gov/clinvar/variation/182430/) without any available experimental evidence reported. Our clinical interpretation (pathogenic) for this variant along with the above experimental information has now been submitted to ClinVar (SUB5321389).

To our knowledge, this study provides the first experimental characterization of the *CHEK2* c.793-1G > A variant, elucidating its impact on splicing.

The proband could receive clinical management based on the NCCN guidelines suggested for *CHEK2* pathogenic variant carriers, such as annual mammogram with consideration of tomosynthesis and breast MRI with contrast at age 40 y.o. [13]. The main limitation of this study was the absence of genetic material from the proband’s mother who had breast and ovarian cancer diagnosis and died at the age of 56. Moreover, testing of the proband’s father was not possible although was requested by our lab.

In conclusion RNA analysis confirmed the disrupting impact of the splice site variant c.793-1G > A in the *CHEK2* mRNA, leading to the definite classification of this variant as pathogenic. We propose that RNA classification should always be conducted wherever an uncharacterized splice site variant is identified in individuals referred for genetic testing.

## Data Availability

All data generated or analyzed during this study are included in this published article. The clinical interpretation of the genomic variant along with the experimental information has been submitted to ClinVar (SUB5321389).

## Abbreviations

ACMG: American College of Medical Genetics and Genomics
AMP: Association for Molecular Pathology
FA: Fancomi Anemia
NCCN: National Comprehensive Cancer Network
NGS: Next Generation Sequencing
RT-PCR: Reverse-Transcription Polymerase Chain Reaction
